# A database of functional traits for spiders from native forests of the Iberian Peninsula and Macaronesia

**DOI:** 10.3897/BDJ.8.e49159

**Published:** 2020-04-30

**Authors:** Nuria Macías-Hernández, Cândida Ramos, Marc Domènech, Sara Febles, Irene Santos, Miquel A. Arnedo, Paulo A.V. Borges, Brent C. Emerson, Pedro Cardoso

**Affiliations:** 1 Island Ecology and Evolution Research Group, IPNA-CSIC, Tenerife, Canary Islands, Spain Island Ecology and Evolution Research Group, IPNA-CSIC, Tenerife Canary Islands Spain; 2 Laboratory for Integrative Biodiversity Research (LIBRe), Finnish Museum of Natural History, University of Helsinki, Helsinki, Finland Laboratory for Integrative Biodiversity Research (LIBRe), Finnish Museum of Natural History, University of Helsinki Helsinki Finland; 3 cE3c – Centre for Ecology, Evolution and Environmental Changes / Azorean Biodiversity Group and Universidade dos Açores - Departamento de Ciências Agrárias e do Ambiente, Angra do Heroísmo, Terceira, Açores, Portugal cE3c – Centre for Ecology, Evolution and Environmental Changes / Azorean Biodiversity Group and Universidade dos Açores - Departamento de Ciências Agrárias e do Ambiente Angra do Heroísmo, Terceira, Açores Portugal; 4 Department of Evolutionary Biology, Ecology & Environmental Sciences, University of Barcelona, Barcelona, Spain Department of Evolutionary Biology, Ecology & Environmental Sciences, University of Barcelona Barcelona Spain; 5 CRBA: Animal Biodiversity Resource Center, UB, Barcelona, Spain CRBA: Animal Biodiversity Resource Center, UB Barcelona Spain; 6 Department of Animal Biology and Edaphology and Geology, University of La Laguna, Tenerife, Canary Islands, Spain Department of Animal Biology and Edaphology and Geology, University of La Laguna, Tenerife Canary Islands Spain

**Keywords:** Araneae, ecology, forest, morphology, Portugal, Spain

## Abstract

**Background:**

There is an increasing demand for databases including species trait information for biodiversity and community ecology studies. The existence of trait databases is useful for comparative studies within taxa or geographical regions, but there is low availability of databases for certain organisms. Here we present an open access functional trait database for spiders from Macaronesia and the Iberian Peninsula, recording several morphological and ecological traits related to the species life histories, microhabitat and trophic preferences.

**New information:**

We present a database that includes 12 biological traits for 506 spider species present in natural forests of the Iberian Peninsula (Spain) and three Macaronesian archipelagoes (Azores, Madeira and Canary Islands). The functional trait database consists of two sections:

1. individual-level data for six morphological traits (total body size, prosoma length, prosoma width, prosoma height, tibia I length and fang length), based on direct measurements of 2844 specimens of all spider species; and
2. species-level aggregate data for 12 traits (same 6 morphological traits as in the previous section plus dispersal ability, vertical stratification, circadian activity, foraging strategy, trophic specialization and colonization status), based on either the average of the direct measurements or bibliographic searches.

This functional trait database will serve as a data standard for currently ongoing analyses that require trait and functional diversity statistics.

## Introduction

An increasing number of ecological studies are incorporating functional traits of organisms to understand global patterns of biodiversity (Díaz et al. 2015), community assembly (Kraft et al. 2008) and ecosystem functioning (de Bello et al. 2010). Functional traits allow us to understand the ecological role of organisms in the community, based on their features and on how the organisms interact in the ecosystem (Nock et al. 2016). Thus, trait based studies allow for the assessment of ecosystem functioning by using species biological traits as a proxy for the functional characteristics of the community assemblage (de Bello et al. 2010). Changes in the functional composition of species assemblages can be related to losses of ecosystem function (Mouillot et al. 2013).

Functional trait databases can be potentially useful for many scientific fields, helping to address questions in systematics, biogeography, macroecology, macroevolution or community assembly. Trait databases are valuable in phylogenetic comparative studies used for the reconstruction of ancestral morphology (Harmon et al. 2010) and to understand how different traits (i.e. body size) have evolved across phylogenetic clades (Kuntner and Coddington 2019), or to link genes with functions or phenotypes (Edmunds et al. 2015). Furthermore, trait-based studies are beneficial in conservation, helping to detect global change impacts or to predict if certain traits are correlated to extinction risk (*Chichorro et al. 2019*), or even to species invasiveness potential (Nyberg and Wallentinus 2005). Trait variation at intra (Garamszegi and Møller 2010) and interspecific levels (Kazakou et al. 2014) are valuable in comparative approaches or community ecology (Bolnick et al. 2011).

As a measure of the diversity of species niches and their functions, Functional Diversity (FD) has been used to understand how species richness or diversity relates to ecosystem function (Petchey and Gaston 2002, McGill et al. 2006, Petchey and Gaston 2006). It is often seen as reflecting a mechanistic link between taxonomic diversity and ecological processes (Cadotte et al. 2011). Given its implications, the selection of traits to quantify FD must be made with caution. Selected traits should reflect the functioning of species in ecosystems and allow understanding of the connection between FD and the response variable of interest according to individual hypotheses (Nock et al. 2016).

Spiders are among the most diverse and abundant predators in all habitats worldwide, being one of the most species-rich invertebrate orders with more than 48,400 described species (World Spider Catalog 2020). Spiders have been used as models in many ecological and evolutionary studies, including functional diversity studies from global (Cardoso et al. 2011) to regional (Cardoso 2012) scales, and methodological advances in phylogenetic and functional diversity analyses (Cardoso et al. 2014). Under this same framework, we are now building a series of analyses that require trait and FD-based statistics, for which the current compilation of traits will serve as a data standard.

We here present a functional trait database of spiders, selecting morphological and ecological traits related to species life histories, microhabitat and trophic preferences, allowing us to characterize the ecological roles of spiders in biological communities and their use of biotic and abiotic resources.

## General description

### Purpose

Our database includes 12 biological traits related to morphology, dispersal ability, vertical stratification, circadian activity, foraging strategy and prey range for all Iberian (Spain) and Macaronesian (Azores, Madeira and Canary Islands) spiders collected during several projects (see funding details in the acknowledgements section). A total of 506 species are included (364 from the Iberian Peninsula, 31 from Azores, 42 from Madeira and 69 from Canary Islands), representing ca. 25% of all acaronesian and Iberian species. The data were collected directly from specimens (i.e. direct morphological measurements), from the field (i.e. vertical stratification and circadian activity), and from scientific publications (i.e. dispersal ability, foraging strategy and prey range).

## Sampling methods

### Study extent

Iberian Peninsula and Macaronesian archipelagoes. In the Iberian Peninsula, spider communities were sampled from white-oak (*Quercus* L.) woodlands across the Spanish National Parks Network: 4 plots in Picos de Europa, 2 plots in Ordesa and Monte Perdido, 2 plots in Aigüestortes i Estany de Sant Maurici, 2 plots in Monfragüe, 4 plots in Cabañeros and 2 plots in Sierra Nevada (Crespo et al. 2018). In the Macaronesian archipelagoes, spiders were collected from natural laurel forest distributed as follows: 6 plots in Pico and 10 plots in Terceira (Azores) (Malumbres-Olarte et al. 2019), 12 plots in Madeira (Malumbres-Olarte et al. 2020), 10 plots in Tenerife and 7 plots in La Gomera (Canary Islands). Morphological measurements were taken directly from all species of spiders collected using the standardized COBRA (Conservation Oriented Biodiversity Rapid Assessment) (Cardoso et al. 2009) sampling protocol.

## Geographic coverage

### Description

Iberian Peninsula (Spain) and Macaronesian archipelagoes (Azores, Madeira and Canary Islands).

### Coordinates

28.121925 and 43.17771 Latitude; -28.422854 and -6.0641 Longitude.

## Taxonomic coverage

### Description

Araneae. The database contains 506 species, accounting for 39 families of spiders. Some morphospecies or cryptic species identified by DNA as different lineages (see Crespo et al. 2018) were also recorded. Taxonomic changes were checked and updated according to the World Spider Catalog (World Spider Catalog 2020).

### Taxa included

**Table:** 

Rank	Scientific Name	Common Name
order	Araneae	spiders

## Traits coverage

This dataset contains six morphological traits, four ecological traits and two other traits related to origin and dispersal ability.

The database consists of two sections:

1. individual-level data for 6 morphological traits (total body size, prosoma length, prosoma width, prosoma height, tibia I length and fang length), based on direct measurements of 2844 specimens of 506 spider species; and
2. species-level aggregate data for 12 traits (the same 6 morphological traits as the previous section plus dispersal ability, vertical stratification, circadian activity, foraging strategy, trophic specialization and colonization status), based on either average direct measurements or bibliographic search.

Morphological traits, vertical stratification and nocturnality were extracted from the samples whenever possible. The combination of sampling methods provides us with direct information on the vertical stratum preferred by the species, from lower to higher stratification: pitfall traps (ground-dwelling species), vegetation sweeping (low vegetation species), active aerial searching and foliage beating (arboreal species). The time of day when species were collected (diurnal vs. nocturnal sampling) provides information about their circadian activity, although this can be biased given the methods used and should be used with caution. Species collected only in pitfall traps did not have such data, with information being extracted from the literature or based on our own field experience instead.

For morphological data, specimens were selected randomly from the available field collections and the measurements were made using a binocular stereoscope. All measurements are given in millimeters (mm). Each morphological trait was measured, whenever possible, for 10 individuals of each species, 5 females and 5 males. For some species only one individual of each sex (the specimen with median body size) was selected for measuring morphological traits other than body size.

In the second section, aggregate data at species level were taken as the average (by sex and total average) of the previous measures for morphological data when available, or from the literature when no individuals of a species could be measured. Additionally, the minimum and maximum ranges of body length for each sex were included. For vertical stratification and circadian activity, we calculated an index based on the collection data or, if not available, using the literature or personal knowledge of species. Other measures were taken from the literature, or often from personal experience with the species in the field.

1. Morphological traits (continuous variables) in millimeters (mm):

- Total body length: measurement of the distance between the front of the carapace (without the chelicerae) and the end of the abdomen (without spinnerets).
- Prosoma length: measurement of the maximum carapace length (without the chelicerae).
- Prosoma width: measurement of the maximum carapace width.
- Prosoma height: measurement of the maximum prosoma height (in lateral view).
- Tibia I length: measurement of the retrolateral length of the first tibia.
- Fang length: measurement of the maximum chelicerae fang length.

2. Ecological traits:

- Verticality (continuous variable): information about the vertical stratification (from epigean to arboreal), based on the information provided by the sampling methods with which the species were collected during fieldwork. One value was assigned to each sampling method (from lower to higher level): pitfall (0), sweeping (1), active aerial searching (2), and beating (3). A weighted mean considering the abundance was performed and this was divided by 3 to rescale the values to a range from 0 to 1. Lower values indicate ground-dwelling species, while higher values indicate arboreal species.
- Nocturnality (continuous variable): information about circadian activity (nocturnal/diurnal) of the species. Diurnal species were recorded as 0, nocturnal species as 1, and for species that can be either diurnal or nocturnal we took a value based on the proportion of individuals of each species active at night during sampling (we divided the number of specimens collected during the night by their total abundance). Values ranged from 0 to 1, with lower values indicating diurnal species and higher values indicating nocturnal species.
- Foraging strategy (eight binary variables; 0 no web - 1 web builders): information about the method of hunting or type of web the species builds. For the web-building species: capture web, sensing web, tube web, sheet web, space web and orb web (more than one feature can be true). For hunting species: ambush hunter and active hunter. The classification was usually made at genus level following the criteria described in Cardoso et al. (2011).
- Trophic specialization (binary variable): information about prey range: stenophagy (specialist species recorded as 1), and euryphagy (generalist species recorded as 0). The classification was generally made at genus level following the criteria described in Cardoso et al. (2011).

3. Other traits:

- Colonization status (categorical variable): for the species distributed in the Macaronesian archipelagoes, information about their origin was included: (E) endemic to the archipelago; (N) native non-endemic; and (I) introduced; according to the Canary Island (Macías-Hernández 2010), Madeira (Cardoso and Crespo 2008) and Azores (Cardoso et al. 2010) spider checklists. The origin status of some species from the Canary Islands was modified according to our knowledge of the species distribution and it will be updated in the public database "Banco de Datos de Biodi­versidad de Canarias" (http://www.biodiversidadcanarias.es/atlantis/common/index.jsf).
- Dispersal: information about dispersal ability was based on ballooning propensity (see also Carvalho and Cardoso 2014). Given the data in Bonte et al. 2003, Bell et al. 2005, Blandenier 2009, we considered as frequent (F) ballooners all species from the families that constituted more than 10% of aerial catches in past studies, in this case Linyphiidae and Araneidae. As occasional (O) ballooners those that made up between 1 and 10%. As rarely (R) or never ballooning species those where ballooning has never been detected or usually represents less than 1% of the catches in published ballooning studies.

## Usage rights

### Use license

Open Data Commons Attribution License

### IP rights notes

All data in the database can be freely used. Please cite this publication or the resource when using newly presented data in your analyses.

## Data resources

### Data package title

Spiders Functional Trait Dataset. Macaronesia_Iberian Peninsula

### Resource link

The data reported in this paper are deposited in the Figshare repository at https://doi.org/10.6084/m9.figshare.8320004.v3

### Number of data sets

2

### Data set 1.

#### Data set name

Individual Spiders traits measurements

#### Data format

.csv

#### Number of columns

19

#### Download URL

https://doi.org/10.6084/m9.figshare.8320004.v3

#### Description

Detailed morphometric measurements dataset including all specimens (2844) of 506 spider species. Measurements in millimeters (mm). Abbreviations: NA: Not Applicable; CRBA: Animal Biodiversity Resource Center; DTP: Dalberto Teixeira Pombo Collection; DZUL: Department of Zoology, University of La Laguna; IPNA-CSIC: Instituto de Productos Naturales (CSIC); MZB: Museum of Zoology, Barcelona; UAc: University of Azores code; UB: University of Barcelona. Observations: (*) specimens whose body size (Total_body_length_mm) is obtained by the sum of cephalothorax plus abdomen; (**) for the columns Prosoma_length_mm and Prosoma_height_mm, measurements with two values separated by -, the first one does not take into account the prosoma protuberance and the second one does take it into account. All column headers (but species) are equivalent to Darwin Core archives from GBIF.

**Data set 1.** 

Column label	Column description
catalogNumber	An unique individual code for each specimen record within the data set or collection
collectionID	An identifier for the collection or dataset from which the record was derived
institutionCode	The name (or acronym) in use by the institution having custody of the object(s) or information referred to in the record
collectionCode	The name, acronym, code, or initialism identifying the collection or data set from which the record was derived
family	Species family
species	Species name
sex	Specimen sex
stateProvince	Province where the specimen was collected
county	County where the specimen was collected
locality	Locality where the specimen was collected
decimalLatitude	Decimal latitude
decimalLongitude	Decimal longitude
Total_body_length_mm	Total body length
Prosoma_length_mm	Maximum carapace length (without the chelicerae)
Prosoma_width_mm	Maximum carapace width
Prosoma_height_mm	Maximum carapace height (in lateral view)
Tibia_I_length_mm	Retrolateral length of the first tibia
Fang_length_mm	Maximum chelicerae fang length
Observations	Special record observations

### Data set 2.

#### Data set name

Aggregate Species Functional Traits

#### Data format

.csv

#### Number of columns

44

#### Description

Summary of morphometric and ecological traits for 506 spider species. Abbreviations: (*) data taken from bibliography, specified in "Citation" column; (§) data from similar species/genera; #N/D: no associated code; NA: not applicable.

**Data set 2.** 

Column label	Column description
species_Lsid	Life Science Identifier from the World Spider Catalog
Species	Species name
Author	Species author
Family	Species family
Coloniz_AZO	Species origin for Azores
Coloniz_MAD	Species origin for Madeira
Coloniz_CAN	Species origin for Canary Islands
Body_length_min_f_mm	Minimum body length female
Body_length_max_f_mm	Maximum body length female
Body_length_min_m_mm	Minimum body length male
Body_length_max_m_mm	Maximum body length male
Body_length_avg_f_mm	Average body length female
Body_length_avg_m_mm	Average body length male
Body_length_avg_mm	Average body length
Prosoma_length_f_mm	Average carapace length female
Prosoma_length_m_mm	Average carapace length male
Prosoma_length_avg_mm	Average of carapace length
Prosoma_width_f_mm	Average carapace width female
Prosoma_width_m_mm	Average carapace width male
Prosoma_width_avg_mm	Average of carapace width
Prosoma_height_f_mm	Average carapace height female
Prosoma_height_m_mm	Average carapace height male
Prosoma_height_avg_mm	Average of carapace height
Tibia_I_length_f_mm	Average of retrolateral length of the first tibia female
Tibia_I_length_m_mm	Average of retrolateral length of the first tibia male
Tibia_I_length_avg_mm	Average of retrolateral length of the first tibia
Fang_length_f_mm	Average chelicerae fang length female
Fang_length_m_mm	Average chelicerae fang length male
Fang_length_avg_mm	Average of chelicerae fang length
Citation	Bibliography from where some measurements were taken if specimens were not available
Dispersal	Dispersal ability as (F)requent, (O)ccasional or (R)are ballooners
Verticality	Vertical stratification (from 0 - epigean to 1 - arboreal)
Nocturnality	Circadian activity (from 0 - diurnal to 1 - nocturnal)
Capture_web	Binary (web used for capturing prey)
Sensing_web	Binary (web used to detect prey)
No_web	Binary (no web used for hunting)
Tube_web	Binary (tube-shaped web)
Sheet_web	Binary (sheet-shaped web)
Space_web	Binary (space, tridimensional-shaped web)
Orb_web	Binary (orb-shaped web)
Ambush_hunter	Binary (hunt by ambushing prey)
Active_hunter	Binary (hunt actively)
Trophic specialization	Prey range (stenophagous or euriphagous)
Observations	Special record observations
